# A refined estimate of the malaria burden in Niger

**DOI:** 10.1186/1475-2875-11-89

**Published:** 2012-03-27

**Authors:** Maimouna Halidou Doudou, Aboubacar Mahamadou, Ibrahim Ouba, Ramatoulaye Lazoumar, Binta Boubacar, Ibrahim Arzika, Halima Zamanka, Maman L Ibrahim, Rabiou Labbo, Seydou Maiguizo, Florian Girond, Julia Guillebaud, Abani Maazou, Thierry Fandeur

**Affiliations:** 1 Programme National de Lutte contre le Paludisme, Niamey, Niger; 2 Unité de Parasitologie, Centre de Recherche Médicale et Sanitaire (CERMES), 634 bd de la Nation, BP 10887, Niamey YN034, Niger; 3 Hôpital National, Niamey, Niger

**Keywords:** Malaria, Niger, Diagnosis, Microscopy, Rapid tests, Slide positivity rate, Seasonality, Spatial variations, Seasonal variations, Incidence, Morbidity

## Abstract

**Background:**

The health authorities of Niger have implemented several malaria prevention and control programmes in recent years. These interventions broadly follow WHO guidelines and international recommendations and are based on interventions that have proved successful in other parts of Africa. Most performance indicators are satisfactory but, paradoxically, despite the mobilization of considerable human and financial resources, the malaria-fighting programme in Niger seems to have stalled, as it has not yet yielded the expected significant decrease in malaria burden. Indeed, the number of malaria cases reported by the National Health Information System has actually increased by a factor of five over the last decade, from about 600,000 in 2000 to about 3,000,000 in 2010. One of the weaknesses of the national reporting system is that the recording of malaria cases is still based on a presumptive diagnosis approach, which overestimates malaria incidence.

**Methods:**

An extensive nationwide survey was carried out to determine by microscopy and RDT testing, the proportion of febrile patients consulting at health facilities for suspected malaria actually suffering from the disease, as a means of assessing the magnitude of this problem and obtaining a better estimate of malaria morbidity in Niger.

**Results:**

In total, 12,576 febrile patients were included in this study; 57% of the slides analysed were positive for the malaria parasite during the rainy season, when transmission rates are high, and 9% of the slides analysed were positive during the dry season, when transmission rates are lower. The replacement of microscopy methods by rapid diagnostic tests resulted in an even lower rate of confirmation, with only 42% of cases testing positive during the rainy season, and 4% during the dry season. Fever alone has a low predictive value, with a low specificity and sensitivity. These data highlight the absolute necessity of confirming all reported malaria cases by biological diagnosis methods, to increase the accuracy of the malaria indicators used in monitoring and evaluation processes and to improve patient care in the more remote areas of Niger. This country extends over a large range of latitudes, resulting in the existence of three major bioclimatic zones determining vector distribution and endemicity.

**Conclusion:**

This survey showed that the number of cases of presumed malaria reported in health centres in Niger is largely overestimated. The results highlight inadequacies in the description of the malaria situation and disease risk in Niger, due to the over-diagnosis of malaria in patients with simple febrile illness. They point out the necessity of confirming all cases of suspected malaria by biological diagnosis methods and the need to take geographic constraints into account more effectively, to improve malaria control and to adapt the choice of diagnostic method to the epidemiological situation in the area concerned. Case confirmation will thus also require a change in behaviour, through the training of healthcare staff, the introduction of quality control, greater supervision of the integrated health centres, the implementation of good clinical practice and a general optimization of the use of available diagnostic methods.

## Background

Niger is a landlocked Sahelian country covering 1,267,000 km^2^. As in many other African countries, malaria remains a leading cause of mortality and morbidity in vulnerable groups, such as children under the age of five years and pregnant women [1]. In 1984, Niger took a timely decision to face up to this situation, by implementing a voluntary national anti-malarial policy. In 2000, its health authorities signed the Abuja Declaration, pledging themselves to halving mortality due to endemic malaria by 2010 and decreasing the malaria burden by 75% by 2015 [2]. With support from the Global Fund and the Roll Back Malaria Partnership (RBMP), Niger has implemented several control strategies to reduce malaria incidence and morbidity in priority groups. These strategies are in line with the Millennium Development Goals, which aim to eliminate malaria by 2030 [3,4].

The interventions integrated into public health policy in Niger include: (i) the adoption in 2005 of the use of artemether-lumefantrine (AL) combinations as the first-line treatment of uncomplicated malaria, replacing conventional anti-malarial drugs; (ii) the adoption in 2005 of intermittent preventive treatment (IPT) with sulphadoxine-pyrimethamine for pregnant women; (iii) the introduction in August 2007 of free health care for children under the age of five years; (iv) the distribution, between 2006 and 2009, of 4,634,706 insecticide-treated nets/long-lasting insecticidal nets. The distribution of these nets has continued, with the aim of achieving universal coverage by 2012; (iv) the management of malaria cases directly at healthcare points. These facilities constituted a network of 1,800 units in 2007 and the number of units has increased steadily ever since, reaching 2,500 by 2010. Similarly, the number of health centres has doubled and there are now 820 centres, each run by a nurse; (v) the introduction in 2008 of the use of artesunate-amodiaquine combinations to combat potential deficiencies in the paediatric form of the AL combination (Co-artesian^®^) and to expand the supply of artemisinin-based combination therapies (ACT); (vi) authorization of the market release of dihydroartemisinin-piperaquine in 2010, and an increase in the number of health workers and physicians assigned to health facilities in the capacity of these centres; and (vii) greater availability of recently introduced malaria treatments and improvements in patient management, with the launch, in 2010, of the Affordable Medicines Facility - malaria, helping Niger to increase the supply of affordable ACT via the public and private sectors, while excluding the use of counterfeit or ineffective anti-malarial drugs [5-8].

However, the beneficial effects of these prevention and control measures remain barely perceptible in Niger, whereas considerable decreases in malaria transmission and incidence have been reported elsewhere in Africa following the introduction of similar control strategies [9,10]. Indeed, over this same period, there has been an unprecedented increase in malaria incidence and the risk of the disease in Niger. The number of malaria cases recorded by the National Health Information System increased by a factor of 5.3 in the space of a decade, from 592,334 cases in 2000 to 3,138,696 cases in 2010, whereas the total population of Niger increased only slightly, by a factor of 1.3, over the same period. Between 2000 and 2010, malaria incidence quadrupled [11,12]. However, this worrying trend was difficult to analyse, because the key performance indicators typically used by the Global Fund and RBMP to evaluate the efficacy and impact of control and prevention programmes, such as the number of people with access to treatment, the number of individuals sleeping under anti-mosquito bed nets, the number of pregnant women receiving IPT and malaria-related mortality were satisfactory [13].

There are, therefore, discrepancies between the considerable financial and human resources that have been mobilized by the health authorities to fight malaria, the satisfactory indicators and the apparent failure of the National Control Programme to reduce the malaria burden in Niger. However, by contrast to the observed increase in malaria morbidity, mortality has continually decreased, from 0.21% to 0.06%, over the same time period. Assuming that the system for recording births and deaths is sufficiently reliable and reproducible from year to year, this implies that the control interventions implemented have at least some effect. It may simply be difficult to determine their impact due to the low accuracy of the basic indicators for malaria assessment. Accurate measurements of malaria burden are essential when planning and advocating malaria control strategies at the national scale [14,15]. In Niger, it is difficult to evaluate the contribution of malaria to disease, because incidence estimates are still largely based on clinical diagnosis, which overestimates the number of malaria cases [16]. The use of biological diagnosis methods for the confirmation of cases of suspected malaria has thus become a key issue in the planning of control activities and disease management in Niger.

A large cross-sectional survey was carried out in 2009 and 2010, in 13 health districts from seven regions of Niger, to address this issue and estimate the discrepancy between the numbers of suspected and confirmed cases of malaria in febrile patients consulting at healthcare points and treated with an anti-malarial drug. The results highlight inadequacies in the description of the malaria situation and disease risk in Niger, due to the over-diagnosis of malaria in patients with simple febrile illness. They highlight the absolute necessity of confirming all cases of suspected malaria by biological diagnosis methods, as recommended by the WHO. Niger extends over a number of latitudes, resulting in considerable geographic heterogeneity in terms of malaria endemicity and populations at risk of the disease. The systematic confirmation of malaria cases by biological diagnosis methods would have major implications for morbidity and future diagnostic in the context of the malaria control programme in Niger and WHO guidelines.

## Methods

### Study sites

Niger is located in West Africa between 11° and 24°N, and 0° and 16°E. It extends over three bioclimatic zones, with a marked gradient of aridity from the Sudan-type savanna in the south, with an annual rainfall of about 500-600 mm, to the arid pre-Saharan zone in the north, with an annual rainfall of less than 250 mm. The central zone belongs to the Sahelian band, corresponding to the northern limit of endemic malaria, whereas the northern zone has a more variable frequency of malaria and tends to be prone to epidemics of the disease. The density and distribution of populations of *Anopheles *species, such as *Anopheles gambiae *and *Anopheles funestus *in particular, are heavily dependent on the local abundance of rainfall. A monsoon occurs over a four-month period each year, from June to September, with an alternation of dry and rainy seasons, but the abundance and distribution of rainfall are highly erratic. The hottest months are April and May, and the cold period extends from November to February. Malaria is transmitted principally during the rainy season, with substantial variations between regions and years [17,18]. A prospective descriptive cross-sectional survey was undertaken simultaneously in 13 districts of Niger, at public health centres in Agadez (Agadez centre/7.292° and 17.211°, and Dagmanett/7.408° and 17.255°), Arlit (Arlit/7.36° and 18.71°), Gaya (Gaya/3.44° and 11.9°, and Yellou/3.58° and 12.26°), Aguie (Tchadoua/7.45° and 13.55°), Maradi (17 portes/7.105° and 13.505°), Niamey II (Saga/2.14° and 13.47°) Madaoua (Bangui/6.19° and 13.67°), Konni (Konni/5.24° and 13.8°), Kollo (Libore/2.49° and 13.41°), Tera (Gothèye/1.57° and 13.85°), Mirriah (Guidimouni/9.5° and 13.69° and Tirmini/8.8° and 13.77°), Magaria (Sassoumbroum/8.49° and 13.12°) and Zinder (Zinder/8.99° and 13.79°). Each health centre was visited twice during the monsoon, from September to October 2009, and then during the post-monsoon period from April to May 2010. The health districts studied are representative of the seven regions of Niger; Diffa was the only region not included in the selection. One or two primary care facilities were selected per district at random, on the basis of reported malaria incidence in 2008 and from a list established on the basis of the following criteria: (i) the health centres selected had to have a supply of valid RDTs; (ii) cases of malaria had to be routinely reported based on presumptive diagnosis; and (iii) the number of reported cases per 100 inhabitants in previous years, calculated from the population covered by each first-contact facility, had to be close to the average for the district to which the health facilities were attached.

### Sample size

The size of the sample to be screened was calculated from the number of malaria cases reported in 2008 and the estimated prevalence, with a 95% CI and a precision level of 1%. The sampling method was based on the selection of regions and health districts proportionally to their size. For a cluster effect of 2, and an estimated prevalence of 50% during the wet season, the sample size required was estimated at 13,000 cases over the 13 selected districts, with a mean of about 1,000 cases per site. Curves of the change in malaria incidence in recent years showed that the number of cases was 10 times higher in the rainy season than in the dry season. For an estimated prevalence of 5%, a precision of 1% and a cluster effect of 2, the estimated sample size was, therefore, 3,643 cases during the dry season, with a mean of about 280 cases per site. All febrile outpatients presenting at health centres with suspected malaria based on presumptive diagnosis were included in the study according to the national policy, encouraging the biological diagnosis of malaria suspected cases. An anonymous identification number was allocated to each patient.

### Field and laboratory procedures

The technical staff recruited for this survey was selected on the basis of their level of motivation and ability to follow the protocol closely. All had solid experience in field surveys and laboratory experiments, including the preparation of blood smears and thin films. They attended a short training course, during which the principal elements of the protocol and the main objectives of the study were explained. Each member of the technical staff was then assigned to a study site (one agent per health centre) with the necessary materials and remained on-site throughout the duration of the study. During the survey, the agents habitually working at the various health centres continued to work as normal, with no change in their working practices. All patients with suspected malaria were referred to the health officer recruited for the study. Thick smears and thin films were then systematically prepared, and a RDT was sometimes carried out using either Paracheck Pf test (Orchid, 4,937 tests performed during the rainy season) which detects only falciparum malaria, and the AgP.f/Pan (SD, INC., 2,876 tests performed during the dry season) test which detects the various species but does not distinguish between *Plasmodium falciparum, Plasmodium ovale, Plasmodium malariae *and *Plasmodium vivax*. All RDTs were interpreted immediately by the resident health officer, to assess the feasibility of introducing RDT-based malaria diagnosis into routine practice at primary healthcare facilities in Niger. All cases of suspected malaria were then treated with an ACT, in accordance with national guidelines. The smears were fixed on-site with methanol and then transported to the laboratory, together with the RDTs. The blood smears were stained with Giemsa for 30 minutes and were read retrospectively by experienced microscopists at the Centre de Recherche Médicale et Sanitaire (CERMES) and Programme National de Lutte contre le Paludisme (PNLP) facilities. A double-blind quality control was performed on 10% of the slides, selected at random, to evaluate reading error. All the RDTs used in the investigation and some unused tests were transported to the laboratory at the end of the study for quality control of readings and to check the validity of the RDTs. The age, usual place of residence and axillary temperature of each patient were recorded, along with the results of the RDT. Giemsa-stained blood smears and RDTs for individual patients were identified by a unique three-digit code, preserving patient anonymity, and all these materials were transferred to a dry, dust-free environment for long-term storage at the CERMES laboratories.

Ethical clearance was obtained from the National Ethics Committee of Niger and permission to conduct the study in the health facilities was granted by the National Health Authorities.

### Data processing and statistical analysis

Data collected during the seasons of high and low rates of transmission were entered into two separate Excel files and processed for statistical analysis with Stata or SPSS software. Percentages, means and standard deviations were calculated. Differences in proportions were assessed in chi-squared tests. Values of *p *< 0.05 were considered significant. The positivity rate was defined as the percentage of patients with suspected malaria testing positive by microscopy (the "slide-positive rate", with the detection of any asexual malaria parasite) or by immuno-chromatography (the "RDT-positive rate"). The diagnostic value of RDTs and fever were estimated and compared with that of microscopy (gold standard) with specialist online software [19].

## Results

### Febrile patients presenting with malaria-like symptoms at health centres in Niger

The recruitment of a total of 16,640 patients with presumptive malaria from the 13 districts was initially planned, to achieve a precision level of 1%. However, unequal consulting frequencies at the various primary healthcare facilities during the wet season precluded the inclusion of such a large number of patients in this study. Finally, 12,576 patients with clinically suspected malaria were enrolled 9,318 during the rainy season and 3,258 during the dry season. The number of patients recruited, although smaller than anticipated, had only a slight effect on the precision of the survey, which remained close to 1%. The Gaya/Yellou, Arlit, Libore and Gothèye health centres were the least busy, whereas the target sample size was reached at other study sites. Consulting frequency was more homogeneous during the post-monsoon season, with a mean attendance rate of about eight patients/day/site. The mean age (and standard deviation) of the patients included in the study was 7.9 ± 12.5 years [95% CI: 7.8-8.0] during the monsoon season and 7.2 ± 12.7 years [95% CI: 6.8-7.6] in the post-monsoon season. Children under the age of five years were more frequently diagnosed with uncomplicated malaria symptoms (66% and 80% during the wet and dry seasons, respectively) than other age groups (Table 1, left panel). Adults (> 15 years old) and children aged between five and nine years were less frequently diagnosed with uncomplicated malaria and subjects between the ages of 10 and 14 years were the least likely to have malaria-like symptoms. These trends were observed at all sites investigated other than those in the north at Arlit and Agadez, where most of the clinical cases were in individuals over the age of 15 years (48% and 39%, respectively) during the season of high transmission rates, this age group generally not accounting for more than 20% of the cases at other study sites (*p *< 0.001). The differences between the seasons of high and low rates of transmission were weakly significant in children under the age of 10 years, but not in older patients. By contrast, the slide-positive rate varied considerably between seasons (*p *< 0.001), regardless of the age of the patients, but the proportion of patients in each age group varied only slightly at a given period and the observed differences were not significant (*p *> 0.05) (Table 1, right panel).

**Table 1:** Suspected malaria cases and slide positive rate by age group and season

Age-group	Suspected malaria cases		*P-*value	Slide positive rate		*P-*value
	Rainy season 2009	Dry season 2010		Rainy season 2009	Dry season 2010	
0-4 years	66	80	0.03	56.6	9.9	< 0.001
5-9 years	13	4	0.02	65.1	9.5	< 0.001
10-14 years	6	3	> 0.05	64.9	12.1	< 0.001
> 14 years	15	13	> 0.05	51.5	9.3	< 0.001
*P*-value	< 0.001	< 0.001		< 0.1	< 0.8	

### Slide-positivity rate

**Figure 1:**
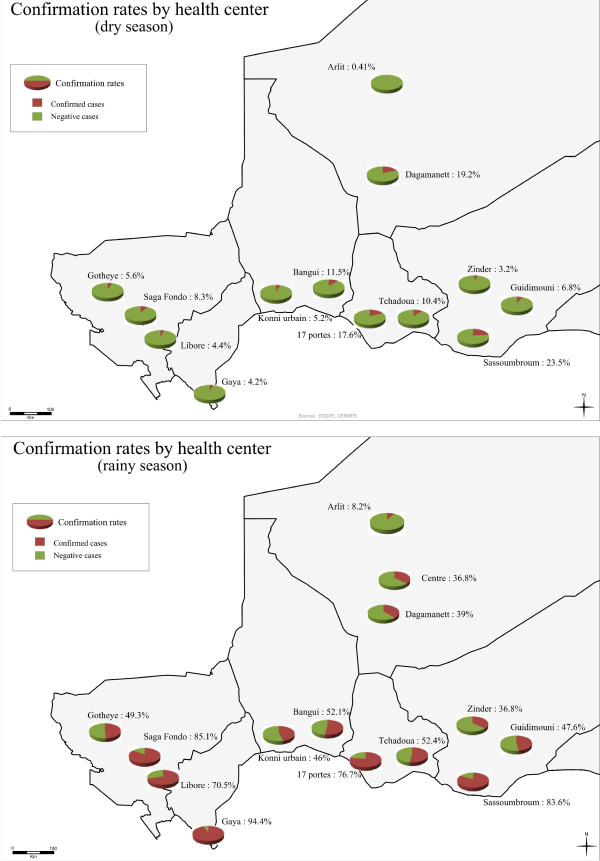
**Maps of Niger showing the geographic distribution of the study areas and the variation of slide-positivity rate during seasons of high (rainy season) and low (dry season) malaria transmission rates**.

The overall frequency of positive slides was 44.7% across Niger, peaking at 57% during the season with high transmission rates and falling to 9.3% during the dry season (Table 2). The proportion of patients with positive slides varied greatly with the location of the study sites. Three main types of situation were distinguished during the monsoon period: 1) high confirmation rates (about 70%) at the Gaya, Saga, Sassoumbroun, 17 Portes and Libore health centres: 2) intermediate rates, close to 50%, at the Tchadoua, Bangui, Gothèye, Guidimoni, Tirmini and Konni sites: 3) low rates (< 40%) in the northern (Arlit and Agadez) and eastern (Zinder) regions. The lowest rate of slide-positivity was that at Arlit, where less than 10% of the clinically suspected cases of malaria were confirmed by microscopy. Similarly, considerable differences between sites were observed during the dry season, with slide-positivity rates generally < 10%, except at Maradi and Magaria, where the confirmation rate was close to 20%. Patient age had little effect on the rate of confirmation by microscopy (*p *> 0.05). By contrast, the season and, thus, the level of malaria transmission had a significant effect on the rate of slide positivity for malaria (*p *< 0.001) (Table 1, right panel). Confirmation rates tended to be highest in the south, where endemicity and transmission levels are also known to be the highest. Malaria confirmation rate followed a north-south gradient and was therefore negatively correlated with latitude. This correlation with latitude was observed only during the rainy season (R2 = 0.61, p < 0.03). No relationship with longitude was observed, regardless of transmission rates. Gaya, the furthest south of the sites studied (latitude 11°9), had the highest confirmation rate, with 94.4% of suspected cases confirmed by microscopy during the wet season. Thus, as in other Sahelian countries, the slide-positive rate in Niger is strongly influenced by bioclimatic context [20,21]. Geographic projections of the slide-positive rate by study site and region are shown in Figure 1. North-south stratification is clearly observed during the monsoon period, when transmission rates are high during the rainy season, but not during the dry season. *Plasmodium falciparum *was found to be responsible for 98.3% of the malaria infections reported during the study, and *P. malariae *was detected in 1.7% of the patients testing positive by microscopy. *Plasmodium ovale *was detected only once, at Konni, and no *P. vivax *infections were observed. *Plasmodium malariae *infections were reported at clustered outbreaks in south-eastern areas of Niger, along the border with Nigeria, at the Sassoumbroum and 17 Portes health centres. Retrospective quality control was carried out for slide reading, on 10% of the slides obtained. A concordance rate of 96.2% was observed in samples collected during the dry season.

**Table 2:** Slide-positive and RDT-positive rates for patients with malaria-like symptoms

Screening	Rainy season 2009	Dry season 2010	*P*-value	Total
Presumptive malaria cases	9318	3258	< 0.001	12576
Slide positive rate	57%	9.3%		44.7%
Presumptive malaria cases	4875	2938	< 0.001	7813
TDR positive rate	42.1%	4.3%		27.9%
*P*-value	0.03	0.15		0.01

**Table 3:** Sensitivity, specificity, positive predictive value (PPV) and negative predictive value (NPV) of RDT

	Rainy season 2009	Dry season 2010	P-values	Total
Sensitivity%	56.4	20.7	< 0.001	52.9
Specificity%	72.4	97.3	< 0.001	85.5
PPV%	67.5	43.3	< 0.001	66.1
NPV%	62	92.5	< 0.001	77.2
P-values	< 0.001	< 0.001		< 0.001

### Frequency of positive RDT results

**Figure 2:**
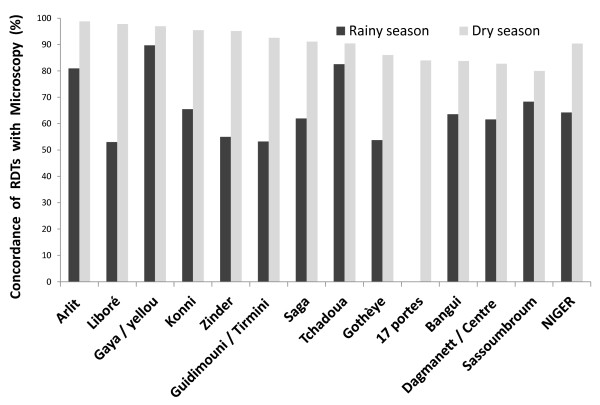
**Seasonal variation and differences between sites in the rate of concordance between RDT and microscopy results**. No RDTs were performed at the "17 Portes" site during the rainy season.

For 7,813 of the 12,576 (62%) patients with a presumptive diagnosis of malaria, a RDT was carried out in parallel with microscopy and read by health workers at the corresponding study site. The RDTs were then transported to the laboratory, for quality control of test reading. A concordance rate of 98% was obtained, demonstrating that healthcare workers were capable of implementing a malaria case detection system based on biological diagnosis at care centres. The RDTs used traditionally in the health system of Niger detect only *P. falciparum*, leaving cases of malaria due to *P. vivax, P. ovale *and *P. malariae *undiagnosed. This strategic choice is supported by the low prevalence of minor species in Niger, the higher cost and lower detection sensitivity of the Combitest, which recognizes multiple *Plasmodium *species. During the investigation, two types of tests were used: the Paracheck Pf and the AgP.f/Pan tests [22]. The raw confirmation data obtained with RDTs are shown in Table 2. Microscopy yielded a significantly (*p *< 0.001) higher proportion of positive results (44.7%) than RDTs (27.9%) for the cases of clinically suspected malaria. This trend was observed during all testing periods. The probability of a positive result was consistently higher for microscopy than for RDTs, with an odds ratio of 1.79 [1.02-3.15] *p *= 0.04 for the monsoon season and of 2.3 [0.7-7.9] *p *= 0.16 for the post-monsoon season. The RDTs used may not be sufficiently sensitive in the conditions tested, but it is also possible that the number of positive cases was overestimated by microscopy, due to undesirable stain precipitation, which may be mistaken for parasites on blood smears. The pan-specific RDTs used here can detect species other than *P. falciparum*, but no infections with any of the minor species were detected. The frequency of positive tests was significantly higher during the wet season (42.1%) than during the dry season, with 4.3% of cases confirmed (*p *< 0.0001). The rate of concordance between microscopy and RDT results varied from 53 to 99%, depending on site and season (Figure 2). The level of agreement was higher during the season of low transmission rates, and considerable differences between seasons were observed, particularly at Libore, Zinder, Tirmini and Gothèye. Taking microscopy as the "gold standard", the use of RDTs at primary healthcare facilities in Niger gave an overall sensitivity of 52.9%, a specificity of 85.5%, a positive predictive value (PPV) of 66.1% and a negative predictive value (NPV) of 77.2%. The specificity and NPV of RDTs were higher after the monsoon, due to the lower incidence of malaria. Conversely, PPV and specificity were highest during the wet season, at 67.5% and 72.4%, respectively. These variations reflect the influence of the seasons and of transmission rates on the diagnostic value of RDTs. These factors should be considered when deciding on the best diagnostic approach for the detection of malaria cases, taking into account both seasonal and spatial variations of malaria incidence. Regardless of the period investigated, the mean rate of concordance between microscopy and RDT results was 74.1%.

### Slide-positive rate and fever

In Niger, the clinical diagnosis of malaria is based essentially on the presence of fever, defined as an axillary temperature of at least 37.5°C. Healthcare workers also take into account other criteria and factors, such as the presence of headache, pain or vomiting and patient age, which may be suggestive of uncomplicated malaria in the absence of fever, to refine their diagnosis. Any recent use of anti-malarial drugs is also noted. During the survey, 31% and 30% of the patients treated for malaria had an axillary temperature < 37.5°C during rainy and dry seasons, respectively. As fever is the principal symptom used for malaria screening, the proportion of patients testing positive for malaria was expected to be lower in the non-febrile patients than in those with fever. However, the fever alone was found of limited value for distinguishing between malaria and other febrile disorders, as the proportions of the included patients testing positive by microscopy were identical in the two groups. Thirty nine% and 6.8% of the febrile patients tested positive for malaria during the rainy and dry seasons, respectively, whereas the corresponding proportions for non-febrile cases were 16.4% in the rainy season and 2.5% in the dry season. These differences are small, but the link between fever and the presence of parasites remains significant during the rainy season (chi^2 ^= 7.9, *p *< 0.01), but not during the dry season (chi^2 ^= 1.1, *p *> 0.05). However, even during the period in which malaria transmission rates were high, diagnosis on the basis of fever seemed to have a low sensitivity (70.4%) and specificity (32.5%), with a low PPV (56.5%). Sensitivity (73%) and specificity (30%) were similar in the dry season, but the presence of fever was even less discriminating (PPV 9.6%). Thus, as demonstrated in previous studies, fever is not clearly correlated with parasite carriage, and the coefficient Q of Yule is negligible (0.07).

## Discussion

All partners involved in fighting malaria in Niger have been committed for a number of years to improving the coverage of populations at risk as concerns basic interventions, in line with the objectives of the RBMP and international recommendations. However, the progress made has not yet resulted in a decrease in malaria levels and morbidity as expected. To tackle this question in the best possible conditions and exploiting recent objective data, a large nationwide survey was carried out aiming at estimating the confirmation rates for the cases declared, most of which were identified on the basis of nonspecific clinical signs of malaria. In a context in which malaria levels are generally declining worldwide, it is essential to have an accurate assessment of the progress made by Niger towards controlling this endemic disease and to identify the trends observed [23,24].

This survey showed that the number of cases of presumed malaria reported in health centres in Niger is largely overestimated, with almost half the patients treated for malaria during the rainy season not carrying parasites. These findings have important implications. Firstly, as concerns individuals, many patients are not receiving the most appropriate treatment. Secondly, at community level, the resulting over-consumption of drugs is very expensive and leads to unnecessary drug pressure favouring the emergence of resistance. According to the WHO, presumptive diagnosis is a widespread practice throughout Africa. Almost 80% of African countries confirm no more than 10% of infections, whereas more than half the cases, if not more, reported in Asia are confirmed. This difference between geographic zones may be accounted for, at least in part, by the higher rates of transmission in Africa, necessitating the mobilization of greater human and financial resources [12,23].

These observations also raise questions about the precision of the malaria indicators used in Niger. The number of cases, divided by population size, provides information about the intensity of endemicity, making it possible to follow the impact of interventions. However, it is clear that the use of presumptive diagnosis statistics in Niger does not provide a sufficiently precise estimate of malaria incidence, particularly as free care and improvements in health cover have led to an increase in the number of consultations. In the past, many people with malaria would not have consulted the health services. The excess cases of malaria recorded in recent years may therefore also reflect a distinct improvement in health cover. Indeed, the incidence data reported in 2009 (154 per thousand) by the National Health Information System in Niger are similar to those reported for the same period by neighbouring Sahelian countries, notably Mali (191 per thousand) and Burkina Faso (195 per thousand), both of which also confirm very few cases. Senegal is the only country in this region in a better position, with an incidence of malaria only one 10th that in Niger (14.6 per thousand in 2009) [25]. Note that Senegal recently implemented a strategy involving the systematic confirmation of malaria cases before notification and treatment [26]. A more realistic estimate of the malaria situation in Niger may be provided by using a correction factor indexed on the mean rate of confirmation calculated here for the country. Weighting the raw data for 2009 by a confirmation index of 0.45 (for microscopy) or 0.29 (for RDTs), gives more realistic estimates of malaria incidence in Niger of 59 and 42 per thousand in 2009. However, this weighted incidence is still high, clear evidence of the progress required for Niger to join the group of African countries that have managed to decrease malaria levels significantly through the implementation of appropriate control measures (Botswana, Cap Verde, Eritrea, Madagascar, Namibia, Rwanda, Sao Tome, Senegal, South Africa, Swaziland, Zambia and Zanzibar, United Republic of Tanzania) [23]. However, these countries are not easily comparable in terms of health, geographic and socio-economic conditions. Niger has a low human development indicator and a high birth rate. The zero to four years age group, which is the most susceptible to malaria, accounts for 20% of the population in Niger, and the fecundity rate of adolescents aged 15 to 19 years is 199 per thousand. For the purposes of comparison, that in Senegal is only 100 per thousand [27].

**Figure 3:**
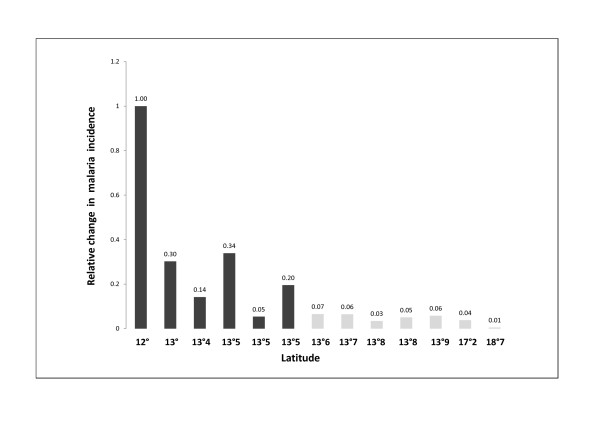
**Relative difference in incidence (rΔinc) with respect to Gaya (the southernmost site) as a function of latitude, during the rainy season when malaria transmission rates were high**. RΔinc is defined by the following equation: rΔinc = SPR Gaya (1-SPR site x)/SPR site x (1-SPR Gaya), where SPR Gaya is the slide-positive rate at Gaya and SPR site x is the slide-positive rate at the other site, x. As Gaya was the site with the highest SPR, all rΔinc values are, by definition, < 1. RΔinc indicates the ratio of the incidence at a given site with respect to that at Gaya, with value of 1 indicating no difference and a value of 0.1 indicating a malaria incidence one tenth that at Gaya.

Niger lies between the latitudes of 11°37' and 23°33' north, and between 16° east and 0°10' west and it covers a total area of 1,267,000 km^2^. Due to its geographic location and its large surface area, climatic and rainfall conditions in Niger are highly diverse. This has resulted in a heterogeneous distribution of *An. gambiae *and *An. funestus*, the principal vectors of malaria, throughout the country. Malaria occurs in an endemic manner, with a seasonal increase in the number of cases during the rainy season. Transmission rates display major temporal and spatial variations, with a gradient of decreasing transmission rates from the south to the north linked to temperatures, rainfall and the availability of surface water. Malaria transmission is generally described by dividing the country into three zones. The extreme south constitutes a zone of stable malaria transmission over more than four months. The north constitutes a zone of instable malaria, with low levels of transmission and a risk of epidemics in certain rainfall conditions. Finally, the zone lying between the north and the south is intermediate in nature, with seasonal transmission limited between August and October [17]. The rate of case confirmation was found to decrease with decreasing endemicity. Thus, in Agadez and Arlit, where malaria is hypoendemic, a mean of 23.4% of slides tested positive in the rainy season, and 9.8% tested positive during the dry season, when transmission rates are low. This observation appears trivial, but the possibility of using slide-positivity rates as a surrogate measurement of incidence provides information about variations in transmission along a south/north line relative to the incidence at the Gaya site (the site furthest south, at 12° north), which is the most heavily exposed [28]. Figure 3 shows variations in the relative incidence of malaria (rΔinc) as a function of latitude in Niger. It was observed that rΔinc calculated from the estimated confirmation rates, based on microscopy, decreased along a latitudinal gradient during the season in which transmission rates were high. This indicator fell from 1 at Gaya to 0.01 at Arlit, corresponding to an incidence two orders of magnitude lower in the most northern part of Niger than in the southern Sudanese zone. The rΔinc values between these two zones were variable. The latitude of 13°5' N, which passes through the towns of Zinder and Niamey, approximately separates zones with different patterns of transmission. This spatial heterogeneity, in addition to the seasonal variations associated with the rainy season, must be taken into account when selecting the most appropriate diagnostic method for the epidemiological situation for malaria in this country. The large difference observed between the numbers of suspected and confirmed cases of malaria introduces a major, non-negligible bias into incidence measurements in Niger. Improvements in the way in which this issue is handled are required. This applies particularly to studies aiming to establish a link between environmental or climatic factors and the risk of malaria and in which estimates are based on historic data concerning the number of suspect cases of fever, indeed it is now known that more than half these cases are of uncertain, but probably viral or bacterial, origin [29]. There is a well-established relationship between exposure to malaria, transmission intensity, age and prevalence. The several studies performed so far in Africa have demonstrated that in areas with intense transmission the bulk of the morbidity caused by malaria mostly concentrated in the younger age groups whereas few cases were reported after the age of 10 years as a result of exposure-dependent acquired immunity. In Niger, the transmission pattern is strongly dependent on climate factors responsible for irregular level of malaria incidence form a year to the next. Thus, climate changes in addition to seasonal variations and fighting interventions would explain periods with low transmission resulting in a loss of immunity in populations, especially those in Northern Niger. Consistent with this hypothesis, we found that significant rates of disease similarly occurred into all age groups, including during into adolescence and adulthood, suggesting that malaria develops at a moderate transmission level in Niger [30,31].

Despite its long history of use for the presumptive treatment of cases of malaria, fever is not a discriminating characteristic [32]. This generalized practice is based on the notion that the disadvantages of unnecessary anti-malarial treatment in patients that do not have malaria are outweighed by the risk of leaving malaria untreated, potentially leading to complications that could rapidly cause the death of the patient. This approach is justifiable in situations of strong endemicity, in which the available anti-malarial drugs are effective, well tolerated and cheap. However, the need to confirm malaria cases by biological diagnosis before notification and treatment, to increase indicator precision and improve the management of patients, is now very clear [33,34]. In Niger, the many cases of presumed but unconfirmed malaria should be considered to be cases of infection of unknown etiology. In the patients concerned, the administration of an anti-malarial drug is of no benefit and, indeed, may actually delay the introduction of an appropriate treatment. Fever cannot be considered an adequate criterion for distinguishing malaria from other infections. In a previous study, Rougemont *et al. *showed that associating the use of fever with that of other criteria, such as the supposed cause and duration of the fever, greatly improved the performance of presumptive diagnosis [35]. However, the use of such a decisional algorithm nationally is not realistic, due to the excessively heavy workload it would create in healthcare structures from the start of the season of high-level transmission, and, in some cases, due to inappropriate clinical practices. The WHO currently recommends the confirmation of all cases of malaria by a biological test before treatment [36]. The malaria transmission in Niger is strongly influenced by epidemiological conditions, due to the large number of latitudes covered by the country. Sensitivity analysis have showed that cost-effectiveness of the diagnosis method depends on several factor among which the malaria prevalence among febrile outpatients is determining. RDTs were shown to be cost-effective at a > 95% confidence level compared with microscopy at malaria prevalence above 20%, which is likely not to be reached in the Northern regions of Niger. From this figure, and provided the question of the performances of RDTs upon exposure at elevated temperature is solved, RDTs are certainly the most appropriate option in the Saharan and Sahelian/Saharan regions, whereas microscopy seems to be more appropriate in the south, below a latitude of 13°5' N. Similarly, RDTs have the potential to be cost-effective in most parts of Niger during the dry season relatively to microscopy [37].

## Conclusion

This survey showed that the number of cases of presumed malaria reported in health centres in Niger is largely overestimated. The results highlight inadequacies in the description of the malaria situation and disease risk in Niger, due to the over-diagnosis of malaria in patients with simple febrile illness. Presented data demonstrate the absolute necessity of confirming cases of malaria recorded in Niger by a biological diagnosis method providing a direct (microscopy) or indirect (RDT) demonstration of the presence of the parasite in the blood before treatment. However, the availability of RDTs and other diagnostic methods at health centres is not sufficient. Only the systematic and effective use of these means will increase the precision of the indicators used in patient care. Pilot studies carried out in 2011, at about a dozen centres in the capital, show that many of these structures had microscopes and RDTs but did not make use of biological detection methods for infections, preferring to rely instead on more rapid clinical diagnosis techniques. Case confirmation will thus also require a change in behaviour, through the training of healthcare staff, the introduction of quality control, greater supervision of the integrated health centres, the implementation of good clinical practice and a general optimization of the use of available diagnostic methods.

## Competing interests

The authors declare that they have no competing interests.

## Authors' contributions

TF and MHD conceived and designed the study, and coordinated study execution. TF wrote the first draft of the paper, prepared tables and figures, and contributed in training health workers. JG, AM, IO and MLI supervised fieldwork. AM, IA and JG also supervised laboratory work and participated in analysing the data; they also contributed in training health workers. RLZ and IO contributed in managing and analysing the data. HZ anf BB contributed in data collection and provided a logistical support. FG did the geospatial information analysis. RL contributed in reviewing the study procedure. SM and AM reviewed the design of the study and the report drafts. All authors have contributed to finalizing the article. All authors read and approved the final manuscript.
